# The effectiveness of stabilization appliance therapy among patients with myalgia

**DOI:** 10.1002/cre2.266

**Published:** 2019-12-02

**Authors:** Tomoyasu Noguchi, Kosuke Kashiwagi, Kenichi Fukuda

**Affiliations:** ^1^ Division of Special Needs Dentistry and Orofacial Pain, Department of Oral Health and Clinical Science Tokyo Dental College Tokyo Japan

**Keywords:** bruxism, myalgia, myalgia, myofacial pain, psychosocial factors, occlusal splints, temporomandibular disorders

## Abstract

**Background:**

The efficacy of stabilization appliance therapy for masticatory muscle pain is debated. Therefore, there are currently no clear usage standards. We analyzed patient factors influencing its efficacy and characterized masticatory muscle pain subtypes to determine appropriate therapy candidates.

**Methods:**

This case series study recruited patients diagnosed with local myalgia or myofascial pain and used variables related to temporomandibular disorders in the analysis. We used temporary appliance to screen patients for sleep bruxism for 2 weeks. Afterwards, we initiated therapy with stabilization appliances. Efficacy was evaluated via tenderness intensity during muscle palpation and the treatment satisfaction score after 2 months of treatment.

**Results:**

We analyzed 62 (91%) patients. Tenderness upon muscle palpation was mitigated in 27 patients. Mitigated tenderness odds ratios were 0.035 for myofascial pain, 0.804 for 15‐item Patient Health Questionnaire scores, and 1.915 for facet length. Thirty‐nine patients expressed satisfaction; satisfaction odds ratios were 0.855 for 9‐item Patient Health Questionnaire scores, 1.606 for facet length, and 4.023 for awake bruxism awareness.

**Conclusions:**

Stabilization appliance therapy is most effective for patients with awake bruxism awareness, local myalgia, long facets, and no psychosocial risk factors.

## 1 INTRODUCTION

According to the U. S. National Institutes of Health, temporomandibular disorder (TMD) is the second most common musculoskeletal problem, after lower back pain, with a prevalence of approximately 5–12% in the United States (http://www.nidcr.nih.gov/DataStatistics/FindDataByTopic/FacialPain). Approximately two thirds of patients with TMD seek treatment, and approximately 15% develop chronic TMD associated with an estimated annual cost of $4 billion in the United States.(Yap et al., 2003) Both joint and masticatory muscle pain are associated with TMD, with masticatory muscle pain occurring more frequently. In Asia, masticatory muscle pain is present in 31.4% of TMD cases, making it the most common TMD subtype.(Yap et al., 2003) Interestingly, another study(Fricton, Kroening, Haley, & Siegert, 1985) found that in at least half of the people with pain in the orofacial area, masticatory muscle pain was the underlying condition. Thus, masticatory muscle pain a clinically important problem.

Stabilization appliance therapy (SAT) has long been used to treat masticatory muscle pain,(Greene & Laskin, 1972) but its efficacy is uncertain; some studies found that it is effective,(Alencar & Becker, 2009; Conti, dos Santos, Kogawa, de Castro Ferreira Conti, & de Araujo Cdos, 2006; Ekberg, Vallon, & Nilner, 2003; Gavish, Winocur, Ventura, Halachmi, & Gazit, 2002; Johansson, Wenneberg, Wagersten, & Haraldson, 1991; Jokstad, Mo, & Krogstad, 2005; Kreiner, Betancor, & Clark, 2001; Manns, Miralles, Santander, & Valdivia, 1983; Nilner et al., 2008; Rubinoff, Gross, & McCall, 1987) and others concluded that it is not.(Dao, Lavigne, Charbonneau, Feine, & Lund, 1994; Truelove, Huggins, Mancl, & Dworkin, 2006) The Japanese Society for the Temporomandibular Joint only cautiously recommends its use as the primary treatment for TMDs.(Yuasa et al., 2013) This caution arises from factors including the inconsistencies between TMD diagnostic criteria from different institutions, the lack of high‐quality research of SAT for masticatory muscle pain, and uncertainty surrounding the causes of masticatory muscle pain.(Raphael et al., 2012; Svensson & Graven‐Nielsen, 2001; Yuasa et al., 2013) Therefore, no clear standards for SAT for masticatory muscle pain exist. In practice, however, SAT is widely used to treat masticatory muscle pain, and some patients experience improvements. Nevertheless, identifying patients who may benefit from SAT is an extremely difficult task. Hence, it is particularly necessary to identify the masticatory muscle pain subtypes that can be effectively treated with SAT.

We analyzed patient factors that might influence the effectiveness of SAT on masticatory muscle pain and aimed to identify the masticatory muscle pain subtypes for which SAT is most appropriate.

## 2 MATERIALS AND METHODS

### 2.1 Patients

This case series study comprised 68 patients (14 men and 54 women; mean age: 48.3 ± 14.4 years) out of 71 patients who presented with orofacial pain at the Tokyo Dental College Suidobashi Hospital between March and December 2016 and who were diagnosed with local myalgia or myofascial pain. We did not recruit patients aged <18 years or those who had moderate or severe systemic disease (i.e., American Society of Anesthesiologists physical status class III or above), loss of posterior support, or temporomandibular joint pain. The exclusion criteria included failure to attend hospital appointments, a deteriorated condition necessitating a treatment switch, and improvements before SAT was started. Written informed consent was obtained from all the participants, and the study protocol was approved by the Ethics Committee of Tokyo Dental College (Ethical Clearance Number 670).

### 2.2 Assessments

Items that are associated with TMD in the existing literature(Türp & Schindler, 2012) were assessed and used in the analysis of patient factors. During the first examination, we assessed tenderness upon muscle palpation. Tenderness intensity at the most tender point during muscle palpation, used as an indicator of the pain threshold, was evaluated on the visual analog scale (VAS). The number of tender areas (both sides), pain‐free mouth‐opening range, awareness of awake bruxism, presence of anterior guidance, open bite, muscle fatigue on waking, torus palatinus or mandibularis, and tongue scalloping or lines on the inner surface of the cheeks were also assessed. The bilateral muscle palpation area was the anterior, middle, and posterior temporalis muscle and the origin, body, and insertion of the masseter muscle. Applying palpation pressure involved using a weight of 1 kgf for 2 s, and prior to palpation, finger pressure was calibrated using an algometer (adjustable spring coil with a small pin touching the examiner's hand when the correct pressure is achieved) in order to standardize the pressure.

To differentiate the types of myalgia, the duration of the pressure was increased to 5 s. Myofascial pain was diagnosed if spreading pain or referred pain was present, and local myalgia was diagnosed if neither was present. Two doctors who have over 5 years of clinical experience in orofacial pain trained by the "Japanese Orofacial Pain Society" made a diagnosis. The other patient factors investigated comprised sleep duration, snoring or apnea, smoking, daily alcohol consumption, daily caffeine consumption, duration of computer usage time, and scores on three self‐administered questionnaires. These questionnaires were the 9‐item Patient Health Questionnaire (PHQ‐9), 15‐item Patient Health Questionnaire (PHQ‐15), and 7‐item Generalized Anxiety Disorder (GAD‐7) scale. These assessments screen for depression, somatization, and anxiety, respectively.

### 2.3 Temporary screening appliances

**Figure 1:**
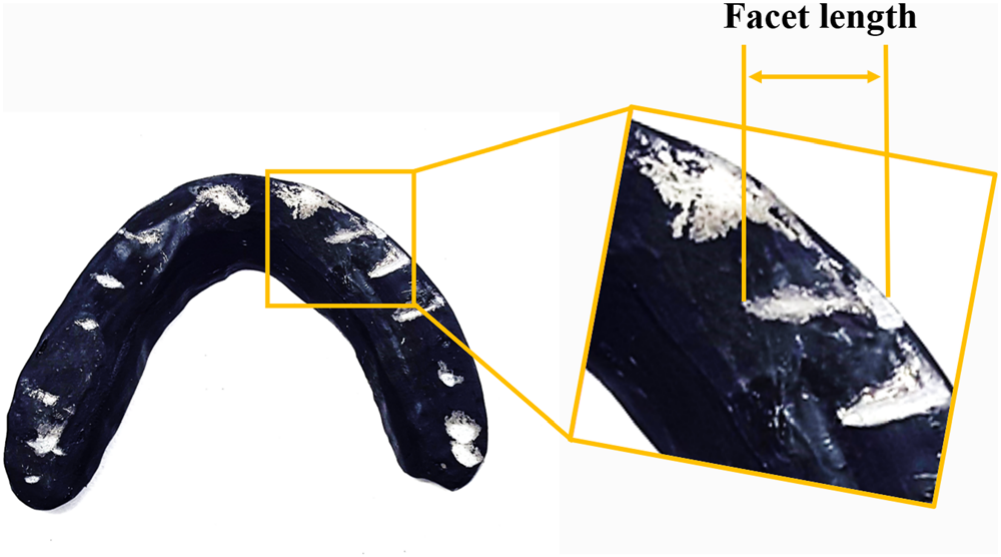
**Temporary screening appliances**. Facets lengths formed by the mandibular canines

Temporary screen appliances were used to screen for facets formed by sleeping. Facets formed by sleeping were observed on the surface of the temporary screening appliances, and the length of the facet was measured (Figure 1). Temporary screening appliances were made from autopolymerizing resin (Facet Resin, GC Corporation, Tokyo, Japan) for nighttime use. This resin was selected because it combines sufficient strength with the appropriate degree of readability for the formation of facets. The appliance's position was adjusted by tapping it until it contacted all teeth equally with canine guidance. After the adjustment was complete, an appliance marker (Facet Resin Marker, GC Corporation) was applied to enable easy observation of the appliance's surface texture. After 2 weeks, the surface texture was assessed, and the lengths of the facets formed by the mandibular canines were measured. However, it should be noted that the effectiveness of temporary screening appliance has not been reported in the past, and detection of bruxism by this is not certain.

### 2.4 Stabilization appliances

After the screening appliance assessment, SAT was initiated. For each patient, an appliance for nighttime use was made to cover the entire maxillary dentition, and the stabilization appliance's position was adjusted in the same manner as the screening appliance's position.(de Leeuw & Klasser, 2013) The adjustment of the stabilization appliance was performed by two dentists. The participants were asked to attend fortnightly appointments at the hospital for stabilization appliance adjustments.

### 2.5 Potential differences between patients with local myalgia and myofascial pain

From the obtained data, the background of patients with myalgia and myofascial pain was investigated, and statistical analysis was performed.

### 2.6 Evaluating the efficacy of SAT

The efficacy of SAT was evaluated in terms of the VAS score indicating the intensity of tenderness during muscle palpation and the treatment satisfaction score 2 months after the start of treatment. A VAS score that was ≥30% lower after treatment than the score before treatment was considered to indicate improvement, and any other score was regarded as a lack of improvement. Treatment satisfaction was self‐assessed as (a) “greatly worsened”, (b) “worsened”, (c) “no change”, (d) “improved”, or (e) “greatly improved”. A score of 1, 2, or 3 was indicated dissatisfaction whereas a score of 4 or 5 signified satisfaction.

### 2.7 Statistical analysis

SPSS version 24 (IBM, Armonk, NY, USA) was used for statistical analysis, and a *p* < .05 was considered statistically significant.

#### 2.7.1 Potential differences for the local myalgia patients and the myofascial pain patients

We compared multiple baseline patient factors in patients with local myalgia and myofascial pain. For the test of normality, the Shapiro–Wilk test was used, and two groups were compared using Mann–Whitney *U*‐test, Student's *t* test, and χ^2^ tests. Logistic regression analysis was performed on all items to obtain odds ratios.

#### 2.7.2 Between‐group comparisons of baseline patient factors

We compared patients who exhibited improvements to those who did not and those who expressed satisfaction to those who did not in terms of multiple baseline patient factors. χ^2^ tests were used to compare the following variables between the two pairs of groups: sex; presence of myofascial pain, anterior guidance, open bite, awareness of awake bruxism, muscle fatigue on waking, torus palatinus or mandibularis, tongue scalloping or lines on the inside of the cheeks, and snoring or apnea; smoking; daily alcohol consumption; and daily caffeine consumption. The Mann–Whitney *U*‐test was used to compare the pretreatment baseline VAS score, number of tender areas, as well as PHQ‐9, PHQ‐15, and GAD‐7 scores. The Student's *t* test was used to compare the baseline sleep duration, duration of computer and smartphone use, pain‐free mouth‐opening range, and facet length in the screening appliance's canine region. These variables were further analyzed with logistic regression analysis to identify predictors associated with improvements in VAS scores and those associated with satisfaction.

#### 2.7.3 Before and after stabilization appliance therapy

Statistical analysis of changes in the number of tender areas, VAS score (intensity of tenderness during muscle palpation), and pain‐free mouth opening range of myalgia at the time of initial examination of all participants 1 and 2 months after starting SAT was performed by a Friedman test.

#### 2.7.4 Between‐group comparisons of patient factors assessed before and after treatment

The Mann–Whitney *U*‐test was used to compare the number of tender areas 2 months after the start of treatment (between both pairs of groups), VAS score at 2 months (between the satisfied and dissatisfied patients), and treatment satisfaction score at 2 months (between the patients who improved and those who did not). Finally, the Student's *t* test was used to compare the pain‐free mouth opening range at 2 months (between both pairs of groups).

## 3 RESULTS

First, this study design is uncoordinated and requires a lot of statistical analysis, so there are Type I errors. Also note that the study power is low due to the small sample sizes.

Of the 68 patients who consented to participate in the study, 3 were excluded because they failed to attend hospital appointments, 2 because their conditions deteriorated so markedly that they were switched to different treatments, and 1 because of an improved condition before SAT was initiated. Thus, the analysis was based on 62 patients (mean age: 48.3 ± 15.2 years). Of them, 12 were men (mean age: 50.5 ±17.1 years) and 50 were women (mean age: 47.8 ± 14.7 years). Overall, there were significant improvements in the VAS score (*p* < .001). There was no significant change in the number of tender areas (*p* = .051) and the pain‐free mouth opening range (*p* = .183; Table 1).

**Table 1:** Before and after SAT

Temporary screening appliances	Pretreatment	1 month after starting SAT	2 months after starting SAT
Pain‐free mouth opening range (mm)			
Median	41	42	41
Interquartile Range	9	8	8
Friedman test	*p* = .183		
VAS score (mm)			
Median	69	52	48
Interquartile Range	31	35	46
Friedman test	*p* < .001*		
Number of tender areas			
Median	6	5	5
Interquartile Range	4	4	5
Friedman test	*p* = .051		

*Note.* Statistical analysis of changes in number of tender areas, VAS score and pain‐free mouth opening range of myalgia at the time of initial examination of all subjects, 1 month after starting SAT and 2 months after SAT started, was performed by Friedman test.

Abbreviations: SAT, stabilization appliance therapy; VAS, visual analog scale.

* *p* < .05.

### 3.1 Potential differences between patients with local myalgia and myofascial pain

There was a difference in the number of tender areas (*p* = 0.029), but there was no difference in other items (Table 2). The odds ratio by logistic regression analysis was 1.295 (Table 3).

**Table 2:** Potential differences between patients with local myalgia and myofascial pain

Variables	Local myalgia	Myofascial pain	*p* value
Number of cases	48	14	
Men/women	11/37	1/13	
Age (year)	47 ± 16	50 ± 12	
VAS score (pretreatment, mm)	63 ± 21	64 ± 25.2	0.52
Tender areas (pretreatment)	5.5 ± 2.3	7.3 ± 3.3	.03***
Pain‐free mouth opening range (pretreatment, mm)	39.8 ± 8.9	39.6 ± 6.8	0.61
Anterior guidance (yes/no)	19/29	9/5	0.1
Open bite (yes/no)	19/29	3/11	0.21
Tooth grinding (during sleep, yes/no)	17/31	6/8	0.61
Awake bruxism (yes/no)	27/21	7/7	0.68
Jaw muscle fatigue (on waking, yes/no)	26/22	8/6	0.84
Torus palatinus (yes/no)	20/28	6/8	0.94
Torus mandibularis (yes/no)	29/19	7/7	0.48
Tongue scalloping (yes/no)	26/22	8/6	0.84
Cheek lines (yes/no)	31/17	6/8	0.14
Snoring or apnea (yes/no)	17/31	4/10	0.63
Smoking (yes/no)	5/43	2/12	0.69
Daily alcohol consumption (yes/no)	13/35	3/11	0.67
Daily caffeine consumption (yes/no)	25/23	10/4	0.2
Sleep duration (hr)	6.1 ± 1.1	6.3 ± 0.9	0.72
Computer usage (hr)	2.1 ± 2.4	1.4 ± 1.6	0.71
Smartphone usage (hr)	1.8 ± 1.8	2.0 ± 1.9	0.61
PHQ‐9 score	8.2 ± 5.6	5.6 ± 4	0.15
PHQ‐15 score	8.5 ± 5.5	12.2 ± 7.7	0.08
GAD‐7 score	7.6 ± 4.7	5.9 ± 4.8	0.24
Facet length (mm)	4.4 ± 2.1	4.8 ± 2.1	0.58

Abbreviations: GAD‐7, 7‐item Generalized Anxiety Disorder scale; PHQ‐9, 9‐item Patient Health Questionnaire; PHQ‐15, 15‐item Patient Health Questionnaire; VAS, visual analog scale.

* *p* < .05 with χ^2^ test.

** *p* < .05 with Mann–Whitney *U*‐test.

*** *p* < .05 with Student's *t* test.

**Table 3:** Logistic regression analysis

Potential differences between patients with local myalgia and myofascial pain			
	Odds ratio	95% CI	*p* value
Tender areas	1.295	1.007–1.666	.044*
Smoking	1.088	0.980–1.208	0.114

* *p* < .05

### 3.2 Improvement versus lack of improvement

Improvement was evident in 27 patients (10 men and 17 women; mean age: 51.0 ±15.1 years) but not in the remaining 35 (2 men and 33 women; mean age: 46.3 ±15.2 years).

#### 3.2.1 Between‐group comparisons of baseline patient factors

The between‐group comparisons, in terms of improvement versus lack of improvement, are shown in Table 4. The improvement rate was significantly higher for men than for women (*p* = .003). Compared with those who did not experience improvements, those who did were significantly less likely to have myofascial pain (*p* = .001), had significantly lower PHQ‐15 scores (*p* < .001), and had significantly longer facets (*p* = .006). Logistic regression analysis of these variables showed that the odds ratios for improvement were 0.035 for myofascial pain, 0.804 for PHQ‐15 scores, and 1.915 for facet length. Sex was not associated with a significant odds ratio (Table 5).

**Table 4:** Patients with improvements versus patients without improvement and satisfied patients versus dissatisfied patients

Variables	Patients with improvements	Patients without improvements	*p* value	Satisfied patients	Dissatisfied patients	*p* value
Number of cases	27	35		39	23	
Men/women	10/17	2/33	0.003*	9/30	3/20	0.508
Age (year)	51 ± 15.1	46.3 ± 15.2	0.227	49.8 ± 15.2	45.8 ± 14.9	0.259
Local myalgia/myofascial pain	25/2	23/12	.001*	30/9	18/5	0.847
VAS score (pretreatment, mm)	61.7 ± 22.3	64.4 ± 20.9	0.515	64.4 ± 21.1	61.2 ± 22.3	0.529
Tender areas (pretreatment)	5.4 ± 2.2	6.3 ± 2.9	0.322	6.0 ± 2.8	5.9 ± 2.3	0.541
Pain‐free mouth opening range (pretreatment, mm)	41.4 ± 6.6	38.3 ± 9.3	0.1	39.6 ± 8.5	40.0 ± 8.3	0.545
Anterior guidance (yes/no)	11/16	16/19	0.695	20/19	7/16	0.182
Open bite (yes/no)	11/16	11/24	0.447	13/26	9/14	0.852
Tooth grinding (during sleep, yes/no)	11/16	12/23	0.601	16/23	7/16	0.574
Awake bruxism (yes/no)	17/10	17/18	0.259	26/13	8/15	0.03*
Jaw muscle fatigue (on waking, yes/no)	15/12	19/16	0.921	23/16	11/12	0.394
Torus palatinus (yes/no)	10/17	10/25	0.48	13/26	7/16	0.964
Torus mandibularis (yes/no)	14/13	16/19	0.631	16/23	14/9	0.212
Tongue scalloping (yes/no)	12/15	22/13	0.149	20/19	14/9	0.639
Cheek lines (yes/no)	16/11	21/14	0.653	23/16	14/9	0.904
Snoring or apnea (yes/no)	9/18	12/23	0.848	14/25	7/16	0.574
Smoking (yes/no)	3/24	4/31	1	4/35	3/20	1
Daily alcohol consumption (yes/no)	10/17	6/29	0.138	11/28	5/18	0.794
Daily caffeine consumption (yes/no)	14/13	21/14	0.521	24/11	11/12	0.293
Sleep duration (hr)	6.3 ± 0.5	6 ± 1.3	0.485	6.4 ± 1.0	5.8 ± 1.0	0.867
Computer usage (hr)	2.6 ± 3.1	2.8 ± 3.3	0.538	2.7 ± 3.2	2.7 ± 3.3	0.547
Smartphone usage (hr)	1.4 ± 1.4	2.0 ± 1.9	0.361	1.8 ± 1.7	1.7 ± 1.8	0.512
PHQ‐9 score	6.6 ± 4.7	8.4 ± 5.5	0.197	6.6 ± 4.1	9.8 ± 6.0	.041**
PHQ‐15 score	6.1 ± 4.1	11.8 ± 6.4	<.001**	8.6 ± 5.9	10.5 ± 6.6	0.501
GAD‐7 score	6.4 ± 4.2	7.8 ± 5.0	0.39	7.1 ± 4.6	7.4 ± 4.9	0.53
Facet length (mm)	5.3 ± 1.8	3.9 ± 2.0	.006***	5.2 ± 1.9	3.4 ± 1.7	<.001***

Abbreviations: GAD‐7, 7‐item Generalized Anxiety Disorder scale; PHQ‐9, 9‐item Patient Health Questionnaire; PHQ‐15, 15‐item Patient Health Questionnaire; VAS, visual analog scale.

* *p* < .05 with χ^2^ test.

** *p* < .05 with Mann–Whitney *U*‐test.

*** *p* < .05 with Student's *t* test.

**Table 5:** Logistic regression analysis

	OR	95% CI	*p* value
Likelihood of obtaining improvement			
Local myalgia/myofascial pain	0.035	0.04–0.344	.004^*^
PHQ‐15 score	0.804	0.683–0.945	.008^*^
Facet length	1.915	1.199–3.058	.007^*^
Likelihood of obtaining satisfaction			
Awake bruxism	4.023	1.063–15.223	.04*
PHQ‐9 score	0.855	0.752–0.972	.016*
Facet length	1.606	1.126–2.291	.009*

*Note.* Logistic regression analysis was performed on items with significant between‐group differences. We treated sex, muscle pain type, and awake bruxism as dummy variables. Others were treated as continuous variables.

Abbreviations: CI, confidence interval; OR, odds ratio; PHQ‐9, 9‐item Patient Health Questionnaire; PHQ‐15, 15‐item Patient Health Questionnaire.

* *p* < .05.

#### 3.2.2 Between‐group comparisons of patient factors assessed before and after treatment

Although there was no significant difference in the number of tender areas on initial examination between patients who improved and those who did not (*p* = .322) 2 months after the start of treatment, the number was significantly lower among the patients who had improved (*p* = .001). There was also no significant difference in the pain‐free mouth opening range on initial examination (*p* = .062), but at 2 months, the range was significantly greater among the patients who had improved (*p =* .019). Satisfaction levels were also significantly higher among the patients who had improved (*p* = .004).

### 3.3 Satisfaction versus dissatisfaction

Satisfaction was expressed by 39 patients (9 men and 30 women; mean age: 49.8 ±15.2 years), and dissatisfaction by 23 patients (3 men and 20 women; mean age: 45.8 ±14.9 years).

#### 3.3.1 Between‐group comparisons of baseline patient factors

The between‐group comparisons, in terms of satisfied versus dissatisfied patients, are shown in Table 4. Compared with the dissatisfied patients, the satisfied patients were significantly more likely to be aware of awake bruxism (*p* = .030), had significantly lower PHQ‐9 scores (*p* = .041), and had significantly longer facets (*p* < .001). Logistic regression analysis of these factors showed that the odds ratios for satisfaction were 0.855 for PHQ‐9 scores, 1.606 for facet length, and 4.023 for awareness of awake bruxism (Table 5).

#### 3.3.2 Between‐group comparisons of patient factors assessed before and after treatment

Although there was no significant difference in VAS scores on initial examination (*p* = .53) 2 months after the start of treatment, VAS scores were significantly lower among satisfied patients than among dissatisfied patients (*p* = .016). There was also no significant difference in the number of tender areas on initial examination (*p* = .542), but at 2 months, the number among satisfied patients was significantly lower (*p =* .016). There was no significant difference in the pain‐free mouth opening range at the initial examination (*p* = .529) or at 2 months (*p* = .546).

## 4 DISCUSSION

TMD is broadly divided into muscle pain and joint pain, with myalgia, tendonitis, myositis, and spasms categorized as muscle pain. Under Diagnostic Criteria for TMDs classifications, myalgia is further classified into local myalgia, myofascial pain with spreading pain, and myofascial pain with referred pain. In this study, we divided myalgia into local myalgia and myofascial pain with spreading or referred pain.

Muscle pain treatments can include patient education, cognitive behavioral therapy, physiotherapy, SAT, and pharmacotherapy.(Klasser, Greene, & Lavigne, 2010) Recently, the American Association for Dental Research has strongly recommended that patients with TMD should initially receive conservative, reversible, and evidence‐based treatments unless they exhibit contraindications (http://www.hotetsu.com/s/doc/aadr2.pdf). Although appliance therapy is regarded as comparatively noninvasive, its efficacy remains unestablished, and it is debatable whether SAT and other treatments that include physical intervention are appropriate. Therefore, at present, the first choice of treatment for TMD is conservative treatment such as self‐massage, and the use of SAT is not recommended. In this study, SAT helped improve myalgia as a result, but we do not recommend SAT as the first choice. However, it may be useful as an auxiliary option for myalgia treatment.

Candidate selection for TMD must consider the distinction between muscle and joint pain. Clinicians should also divide muscle pain into local myalgia and myofascial pain and further investigate the appropriate treatments for each. Adverse events and medical expenses arising from inappropriate TMD treatment selections are important issues. Therefore, detailed examinations of patient factors, including psychosocial factors, are indispensable when selecting treatments for masticatory muscle pain. Our results suggest that in addition to traditional TMD examinations, indicators for muscle pain subtypes treatable with SAT include awareness of bruxism, appliance‐based bruxism subtype screenings, muscle pain subtype diagnoses, and psychosocial factor screenings.

SAT is a long‐established treatment that is regarded as excellent by some clinical practitioners. However, some studies have suggested that the benefits of SAT do not surpass those of placebos and only offers short‐term relief for muscle pain.(Klasser et al., 2010) However, our results show that the VAS scores of all participants decreased significantly. This result suggests that SAT contributed to an increase in the pain threshold and there may be an effect of pain relief in masticatory muscles.

Because TMD arises from complex interactions between various factors, the diagnostic criteria for control group patients with TMD should ideally be like those for treatment group patients regarding Diagnostic Criteria for TMDs Axes I and II (https://ubwp.buffalo.edu/rdc-tmdinternational/tmd-assessmentdiagnosis/dc-tmd/). However, ensuring such consistency is extremely difficult. Therefore, this study did not include a control group. Instead, we categorized patients based on whether the treatment ameliorated their symptoms and then analyzed differences in patient factors between those categories. Hence, a limitation of this study is that we did not investigate placebo effects. Furthermore, the patients might have spontaneously healed, so our evaluation of therapeutic efficacy might have included placebo effects and spontaneous healing.

### 4.1 Awareness of awake bruxism

Self‐reported awake and sleep bruxism are both risk factors for TMD.(Huhtela et al., 2016) Awake bruxism includes both tooth clenching and light tooth contact. Habitual tooth contact is evident in 52.4% of patients with TMD and is associated with a 1.944‐fold increased probability of continuance or worsening of TMD pain.(Sato et al., 2006) Self‐reported tooth contact or tooth clenching is also a risk factor for facial pain.(Glaros & Williams, 2012) In this study, 54.8% of the participants were aware of having awake bruxism. Our finding that awareness of awake bruxism was significantly more common among satisfied patients than among dissatisfied patients was unexpected, given that the stabilization appliances were only worn at night. Because many of the highly satisfied patients were aware of having awake bruxism and showed long facets in screening appliances, the highly satisfied patients might have had both awake and sleep bruxism. Reissmann et al.(Reissmann et al., 2017) reported that patients who were aware of having both awake and sleep bruxism were at an increased risk of developing painful TMD. If the stabilization appliance positively influenced sleep bruxism perhaps by relieving muscle fatigue and physiological stress caused by grinding, patients may have been highly satisfied due to a reduced risk of painful TMD. Because we did not investigate biological responses during sleep, further research is needed to test this hypothesis.

### 4.2 Types of sleep bruxism

Sleep bruxism may cause muscle pain, and the use of stabilization appliances to control sleep bruxism has been widely reported.(Klasser et al., 2010) Recently, however, many authors have questioned whether sleep bruxism causes muscle pain because several studies comparing patients with masticatory muscle pain to a control group found that muscle activity during sleep was actually greater in the control group.(Lavigne, Rompré, Montplaisir, & Lobbezoo, 1997) Moreover, other studies have shown that muscle activity decreases when muscle pain is present.(Murray & Peck, 2007) These findings have also cast doubt on the efficacy of SAT.(Svensson & Graven‐Nielsen, 2001) However, no previous studies have assessed the efficacy of SAT after classifying patients with muscle pain according to sleep bruxism subtypes, so further research is required.

Polysomnography is the standard technique for analyzing sleep bruxism, but it is expensive, requires specialist expertise for analysis and diagnosis, and poses difficulties for patients due to the long period of restraint required and the altered sleep environment. Therefore, we used screening appliances in this study. Although screening appliances do not provide extensive information, they suffice for sleep bruxism screening and, compared with polysomnography, are less burdensome for patients. As the number and timing of events associated with sleep bruxism vary from day to day, another advantage of using screening appliances is that the results reflect the entire period of use.

We found that the patients with improved VAS scores and those who were satisfied had significantly longer facets than those with no improvement and those who were dissatisfied, respectively. Screening appliance examinations may be important tests for whether patients have muscle pain subtypes for which SAT may be effective. Long facets are associated with grinding‐type bruxism. During tooth grinding, tooth engagement imposes excessive loads on the muscle, but the stabilization appliances counteract the effects of tooth engagement by reducing lateral pressure and mitigating negative effects on the musculature. In a comparative study of patients with different types of sleep bruxism, Yoshimi et al.(Yoshimi, Sasaguri, Tamaki, & Sato, 2009) found that muscle activity was most intense during grinding. This suggests that decreasing the force imposed during grinding relieves muscle fatigue, which might have influenced the VAS improvements and treatment satisfaction scores that we observed. Furthermore, myalgia is believed to be caused by impaired blood flow due to excessive muscle use and sympathetic reflexes.(de Leeuw & Klasser, 2013) Although muscle stress reduction and psychological stress may lead to blood flow improvements and positive health outcomes by promoting bruxism by stabilization appliances, research is needed that include a heart rate variability analysis, near‐infrared spectroscopy, electromyography, and accelerometer use.

### 4.3 Type of muscle pain

Patients who had significantly improved VAS scores showed more local myalgia than myofascial pain. Myofascial pain is characterized by deep or spreading pain and is associated with factors such as impaired peripheral blood flow (i.e., hypoxia), the pain‐inducing action of growth factors, and hypersensitivity of the sympathetic nervous system that can increase sensitivity to palpation or tenderness to pressure, sometimes with referred pain.(Maekawa, Clark, & Kuboki, 2002) In other words, central sensitization or peripheral sensitization may be involved. The difference in the tender areas between local myalgia and myofascial pain in this study result also suggests that sensitization is involved.

TMD is categorized as a type of functional somatic syndrome, alongside fibromyalgia and somatic symptom disorder,(Henningsen, Zipfel, & Herzog, 2007) and the diagnostic criteria for myofascial pain and fibromyalgia have many similarities.(Wolfe et al., 1990) If TMD‐associated myofascial pain is truly like fibromyalgia, then SAT may be less effective for myofascial pain than for local myalgia, as shown in our results. If local myalgia is caused simply by muscle fatigue, then SAT should be more effective for local myalgia than for myofascial pain. However, the existence of psychosocial risk factors and the involvement of central hyperalgesia, such as that observed in fibromyalgia, must be considered when managing myofascial pain.

### 4.4 Psychosocial risk factors

Psychosocial factors influence the risk of developing chronic lower back pain and other musculoskeletal disorders,(Hasenbring, Hallner, & Klasen, 2001) including chronic TMD.(Harper, Schrepf, & Clauw, 2016; Slade et al., 2016) We found that high PHQ‐9 scores were significantly associated with dissatisfaction with treatment and that high PHQ‐15 scores were associated with VAS scores indicating a lack of improvement.

Depression is thought to be closely association with pain.(Wright et al., 2004) Chronic stress due to factors such as pain causes both depression and hyperalgesia,(Rivat et al., 2010) and depression impairs the function of the descending pain modulatory system.(Stahl, 2002) Screening for depression is regarded as essential in managing chronic lower back pain,(Tsuji, Matsudaira, Sato, & Vietri, 2016) and accumulating evidence indicates an association between musculoskeletal pain and depression. For example, patients with TMD due to muscle pain have higher depression scores than patients with other TMD subtypes, suggesting that depression screenings would facilitate pain management for patients with TMD.(Bertoli & de Leeuw, 2016)

The PHQ‐9 is widely used in Japan as a screening tool, and its use has highlighted the association between pain and depression. High PHQ‐9 scores are significantly associated with higher pain intensities in patients with chronic lower back pain,(Vietri, Otsubo, Montgomery, Tsuji, & Harada, 2015) and 22% of individuals who have been injured for ≥90 days have PHQ‐9 scores indicative of depression,(Zhou & Jia, 2016) signifying that managing pain in patients with high PHQ‐9 scores is difficult. Such patients therefore require the early adoption of a multifaceted approach, as satisfaction is unlikely to be obtained with SAT alone.

Studies on psychosocial stress and TMD have shown that pronounced somatic symptoms represent a strong risk factor for developing TMD.(Fillingim et al., 2013) The PHQ‐15 is also effective for evaluating fibromyalgia severities.(Häuser, Brähler, Wolfe, & Henningsen, 2014) Fibromyalgia and TMD are both forms of functional somatic syndrome,(Henningsen et al., 2007) and because they have many similarities, the PHQ‐15 can also be useful for assessing patients with TMD. Fibromyalgia pain is thought to be neuropathic or central pain rather than nociceptive pain, with pain hypersensitivity as a contributing factor. High degrees of sensitivity to pressure, heat, and pinprick stimulation have also been reported in chronic TMD;(Greenspan et al., 2011; Greenspan et al., 2013) also, central hyperalgesia is thought to be involved. We found that compared with patients whose VAS scores indicated improvements, those whose VAS scores indicated no improvement (i.e., patients with low pain thresholds) had significantly higher PHQ‐15 scores and a higher frequency of myofascial pain. The contribution of somatic symptoms and hyperalgesia must be considered when managing myofascial pain in patients with high PHQ‐15 scores because in this study, these patients were less likely to experience improvements compared with patients with myofascial pain who had low PHQ‐15 scores. These patients may need an alternative approach to SAT.

The GAD‐7 is a valuable anxiety screening tool.(Löwe et al., 2008) Although its use may be regarded as essential in managing TMD, we found no significant difference in GAD‐7 scores between the patients who improved and those who did not or between the satisfied and dissatisfied patients.

Our results showed that higher PHQ‐9 scores were associated with lower likelihoods of satisfaction with SAT, whereas higher PHQ‐15 scores were associated with lower likelihoods of experiencing improvements, as reflected in VAS scores. In a study of patients with residual pain following knee surgery, Bierke et al.(Bierke, Häner, & Petersen, 2016) found that scoring ≥10 on both the PHQ‐9 and PHQ‐15 was associated with significantly greater knee pain and a higher likelihood of being dissatisfied with treatment, which is consistent with our findings. Our findings are also consistent with those of other studies that found that various psychosocial risk factors heightened sensitivity to pain and reduced the probability of patients responding to standard treatments.(Ohrbach & Dworkin, 1998)

In TMD management, it is important to evaluate psychosocial risk factors during both the initial examination and the course of treatment and to consider approaches other than conventional treatments such as conservative therapy, SAT, and physiotherapy.

## 5 CONCLUSION

Various masticatory muscle pain subtypes exist. Therefore, detailed examinations of factors, including psychosocial factors, are essential for effectively treating patients with masticatory muscle pain.

Our results suggest that patients aware of awake bruxism and with local myalgia who formed long facets on their stabilization appliances would respond better to SAT than those who have myofascial pain or formed short facets. High PHQ‐9 scores indicate a reduced likelihood of satisfaction with SAT, and high PHQ‐15 scores indicate a reduced likelihood of benefiting from this treatment in terms of VAS scores for tenderness during muscle palpation. Therefore, SAT may be most effective for patients aware of awake bruxism and with local myalgia who form long facets on the stabilization appliances and who lack psychosocial risk factors.
